# Preferences and related factors for postpartum contraception in pregnant women

**Published:** 2013-10

**Authors:** Gülay Yilmazel, Elçin Balci

**Affiliations:** 1 *Department of Public Health, Health School, Hitit University, Corum, Turkey.*; 2 *Department of Public Health, Medical Faculty, Erciyes Universty, Kayseri, Turkey.*

**Keywords:** *Pregnantwomen*, *Postpartumperiod*, *Contraception*, *Preferences*, *Factors.*

## Abstract

**Background:** The postpartum period is a time of transition for a pregnant woman and her new family. In this period many pregnant women are in search about the family planning methods. But contraceptive options differ depending on women’s desires such as cultural and religious believes, partner attitudes, previous contraceptive experiences.

**Objective: **This study was conducted to identify status of using a contraceptive method before pregnancy and the factors associated with preferences of contraception in postpartum period.

**Materials and Methods:** The descriptive research was conducted in a State Hospital March-May 2012 in Turkey. The population of study was formed with 200 pregnant women who applied follow-up pregnant clinics. We took permissions from local authorities and participants. 182 voluntary pregnant women were surveyed. We prepared a 20 item question are form which was asking socio-demographic futures, contraceptives methods before-after delivery and the factors related with using contraceptives after screening literatures related with subject.

**Results: **The 49.5% of women reported that they didn’t use any methods before. There was a significant relation between using contraceptives before pregnancy with the idea of using contraceptive during the postpartum period and receiving contraception counseling during pregnancy (p=0.004, p=0.035 respectively).

**Conclusion:** The 86.4% of pregnant implied that they would use a contraceptive method in postpartum period. IUD was the most preferred method. Status of using contraceptive before and receiving contraception counseling in pregnancy were the effective variables on thoughts about using a contraceptive method. To achieve desired goals for maternal and child health in our country health professionals should be more focused on postpartum contraception in antenatal care programs.

## Introduction

The postpartum period is a time of transition for a woman and her new family, when adjustments need to be made on physical, psychological, and social levels (1). Pregnancy and the birth of a child change a woman’s pirorities, attitudes and lifestyle, sex behavior and use of preferred contraceptive method. During the postpartum period, effective contraception can prevent unintended pregnancy and ensure adequate birth spacing (2-5). 

After a live birth, the World Health Organization (WHO, 2005) currently recommends an interval of 24 months before attempting to become pregnant again (6). Fertility control with using contraceptives is essential to the health and welfare of individuals, families and communities. The first important contribution to the use of contraception to reduce the number of unwanted births, unsafe abortion is the second most important contribution to reduce the morbidity and mortality associated with obstetric (7, 8). Despite all these problems, the worldwide estimation of contraceptive prevalence among 15-49 years of age is 62.7% (9).

In our country, the rate of using any contraceptive method is 73.0% and using effective method is 46% among 15-49 years of age (10). Several forms of contraception are safe and effective for use in the postpartum period. But contraceptive options differ depending on patient desires such as cultural and religious believes, partner attitudes, previous contraceptive experiences, reproduce plans, risks related pregnancy, side effects related with contraceptive methods and perceived risks, prices, convenience, efficiency, individual risks in sexually transmitted diseases (11). In postpartum period many women are in search about the family planning methods and they tend to receiving information about different contraceptive methods from health professionals who took care of their health. This situation is an opportunity for clinicians, nurses and midwives to promote modern methods. 

This study was conducted to determine the status of using a contraceptive method before pregnancy, contraception choices in post-natal period and related factors in pregnant women.

## Materials and methods

This descriptive study was conducted in a State Hospital March-May 2012 in Turkey. The population of study was formed 200 pregnant women applied to pregnant follow-up clinics. Before the research a written permission was taken from the local health authority and from Erciyes University Ethics Committee. Pregnant women were informed about the research and 182 pregnant were surveyed who accepted to join the study without sample selection.

The rate of achieving was 91.0%. The questionnaire form was prepared by the researchers after literature screening which was consist of 20 questions with socio-demographic features, history of previous pregnancy and family planning methods, postpartum contraceptive preferences, reasons for selecting methods. 

**Statistical analysis**

The data were evaluated with the percentage, arithmetic mean, Pearson chi-square and Fisher’s exact test by using the SPSS 11.5 package program. P<0.05 values were considered statistically significant.

## Results

The mean age of pregnant women was 26.57±4.98 (SD) and 50.0% of between 26-34 ages. The 47.8% of pregnant women graduated primary school, 95.6% of didn’t work 62.1% of lived in city center and 64.3% of perceived their economic status as a mid-level. 50.8% of pregnant women reported that they would be the second time mothers, 49.5% of said that they didn’t use any method before pregnancy. Socio-demographic characteristics of pregnant women were given in table I. In table II relation between the various features of pregnant women with thoughts about using a contraceptive method after delivery were shown. It was found no statistical significant relation between thoughts about using postpartum contraceptive method with their ages, levels of education, husbands’ levels of education, duration of marriage and number of births. 

The rate of pregnant women who used a contraceptive method before also thought using a contraceptive method after the birth (42.9%) were higher than the rate of pregnant women (29.1%) who didn’t use any method before then consider using methods after the birth. The relation between thoughts about using contraceptive methods in postpartum period with the status of using prenatal contraceptive were significant (χ^2^:6.514, p=0.004). 

Pregnant women who didn’t take contraception counseling thought using a contraceptive method during the postpartum period (50.5%). There was a statistically positive significant relation between taking contraception counseling during pregnancy with using postpartum contraceptive method (χ^2^:7.984, p=0.035). The reasons for wanting and not wanting to use postpartum contraceptive were shown in table III. Pregnant women of 39.6% stated that they wanted to use contraceptives for prevention future pregnancies, 31.9% of for child spacing. The 13.6% of women declared they didn’t want to use a contraceptive method during the postpartum period for some reasons.

Table IV showed the preferred postpartum contraceptive choices. IUD was the most preferred postpartum contraceptive method by pregnant women. The 24.2% of women said that their husbands would use condom. The third preferences of women were combine oral contraceptives. Table V showed factors that influenced contraceptive choices in these women.

**Table I:** Socio-demographic characteristics of pregnant women

**Socio-demographic characteristics (n=182)**		**N**	**%**
Age			
	17-25	80	44.0
	26-34	91	50.0
	35 age and over	11	6.0
Education level			
	Primary	87	47.8
	Secondary	44	24.2
	High school/ university	51	28.0
Job			
	Working	8	4.4
	Non-working	174	95.6
Spouses education level			
	Primary	60	33.0
	Secondary	43	23.6
	High school/ university	78	43.4
Place of residence			
	Province	113	62.1
	District	35	19.2
	Town	14	7.7
	Village	20	11.0
Perceived Economic status			
	Good	65	35.7
	Mid	117	64.3
Number of pregnancies			
	1	55	30.2
	2	56	30.8
	3	45	24.7
	4 and above	26	14.3
Any of method used before			
	Used	92	50.5
	Not used	90	49.5
Total		182	100.0

(Age mean: 26.57±4.98 years)

**Table II:** Correlation between the various features of pregnant women with thoughts about using a contraceptive method after delivery

**Features**			**Thoughts about using a contraceptive method**		**χ** ^2^	**p**
			**Yes**	**No**		
Age					1.712	0.310
	17-25		60 (33%)	22 (12.1%)		
	26-34		65 (35.7%)	24 (13.2%)		
	35+		6 (3.3%)	5 (2.7%)		
Level of education					2.747	0.109
	Primary and below		63 (34.6%)	24 (13.2%)		
	Secondary		30 (16.5%)	14 (7.7%)		
	High school/ university		42 (23.1%)	9 (4.9%)		
Husband education level					5.526	0.457
	Primary and below		43 (23.6%)	13 (7.2%)		
	Secondary		30 (16.5%)	17 (9.3%)		
	High school/ university		62 (34.1%)	17 (9.3%)		0.097
Duration of marriage					7.897	
	0-5 year		63 (34.6%)	31 (17.1%)		
	6-10 year		41 (22.5%)	14 (7.7%)		
	11 years and over		28 (15.3%)	5 (2.8%)		
Number of births					6.306	0.184
	0		38 (20.9%)	19 (10.4%)		
	1		54 (29.7%)	15 (8.2%)		
	2		27 (14.8%)	12 (6.6%)		
	3+		16 (8.8%)	1 (0.6%)		
Status of contraceptive use before					6.514	0.004
	Used		78 (42.9%)	14 (7.7%)		
	Not-used		53 (29.1%)	37 (20.3%)		
Receiving contraception counseling					7.984	0.035
	Yes		39 (21.4%)	5 (2.7%)		
	No		92 (50.5%)	46 (25.3%)		

**Table III:** Reasons for wanting and not wanting to use contraceptives

**Reasons for wanting to use a contraceptives [n=157 (86.4%)]**			**N**	**%**
For child spacing			58	31.9
Prevention of future pregnancies			72	39.6
Doctors/nurses advice			21	11.6
Friends use it			6	3.3
**Reasons for not wanting to use contraceptives [n=25 (13.6%)]**				
		Desire for more children	9	4.9
		Faith/religion	5	2.7
		Previous negative experiences with contraceptive	5	2.7
		Husband object to it	6	3.3
Total			182	100.0

**Table IV:** Respondents preferred postpartum contraceptive choice

**Preferred method (n=157)**	**N**	**%**
Intrauterine contraceptive device (IUCD)	56	35.7
Men condoms	38	24.2
Combine Oral Pills (COP)	32	20.4
Tubal ligation	25	15.9
Spermicide, Injectable	6	3.8
Total	157	100.0

**Table V:** Factors that influence contraceptive choices

**Influencing factors (n=157)**	**N**	**%**
Husbands	55	35.0
Doctors/ Nurses	45	28.7
Friends	31	19.7
Pharmacist/ Chemist	16	10.2
Media	10	6.4
Total	157	100.0

## Discussion

The prevalence of using contraceptive methods among pregnant women was 50.5% (Table II). Studies held in Istanbul, it was found 47.6%, in America was 80.0% and in Nigeria was 35.5% (2, 7, 11). The result was pleasing but insufficient condition for awareness of contraceptive methods among pregnant women. Pregnant women should be having effective contraceptives. Because effective contraceptives prevent gynecological complications, unwanted pregnancies, voluntary abortus. 

Also it is important for lengthening birth intervals. Therefore, clinicians, particularly nurses and midwives have great responsibility for giving contraception counseling services during the prenatal period. A study which was conducted in Nigeria it was found a significant relation between thoughts of using contraceptive method with ages, levels of education, husbands level of education, number of births and taking contraceptive counceling. Also it was determined that duration of marriage didn’t affect the thoughts of contraceptive using (7). In Florida it was showed no relation between ages, number of births, levels of education with using of postpartum contraceptive method. 

But a significant relation showed between using contraceptive before and taking contraceptive counsels during pregnancy with thoughts of using postpartum contraceptive methods (12). In this study there was no relation between pregnant women’s thoughts of using pospartum contraceptive methods with their ages, levels of education, husband’s level of education, number of births and duration of marriage. 

But we found a statistical significant relation between using contceptive before pregnancy and receiving contraceptive counseling with thoughts about using postpartum contraceptive method (p=0.004, p=0.035) (Table II). Being satisfied with the method used before pregnancy can be effective on the idea of using the method; during the postpartum period. It was surprised that women who said they didn’t receive contraception counseling during their pregnancy reported that they would use a contcaceptive method in postpartum period. Unmet needs for contraception can be lead pregnant women unconsciously seeking any method themselves. 

Therefore, all pregnant women in follow-up clinics questioned whether they receive family planning counseling before. Women during pregnancy may be in a quest for knowledge about contraceptive methods using after the birth. Health care professionals were the most reliable source which will provide this information to pregnant women. If pregnant women didn’t have their information needs about contraception from health care providers for close these needs can turn to diverse source (friends, pharmacy, media, etc.). 

This is a missed opportunity in the promotion of modern contraceptive methods. Missed opportunities should be assessed on the next follow-up of pregnant and effective consulting services should be given to pregnant women and their husbands. Among pregnant women admitted to hospitals and clinics in Sub-Saharan Africa, South Africa and Latin America planning to use a contraceptive method after delivery was found to be between 40-96% (13-15). In this study, 86.4% of pregnant women stated that they would use a contraceptive method after the birth. The 39.6 of pregnant women considered using a contraceptive method in order to prevent future pregnancies (Table III). 

In a study conducted in Malatya 32.9% of women, in Western Europe 19.0%, in the Czech Republic 18.8% thought to use IUD in postpartum (16-18). In our study, 35.7% of women stated that their contraceptive choice were IUD in the postpartum period (Table IV). Reasons such as being safe, failing no load to users and using for a long time can contribute to IUD to be more preferred than other methods. Also husbands’ choices influenced the using contraceptives in our study (35.0%). In El Salvador and the United States results are consistent with obtained from our study (19, 20). 

Husbands’ attitudes may positive or negative impact on the use and choice of postpartum contraception. Women may not be having full control over their own sexual health and reproductive lives. Because our community is a patriarchal society, it is important that counseling programs should be in given specially husbands of women in antenatal care and they should be integrating to this program (Table V). 

As a result, an important part of the pregnant women (86.4%) stated that they would to use a contraceptive method after the birth. IUD was the most preferred method by pregnant. The rate of contraception counseling during pregnancy was (21.4%) quite low. Husbands of pregnant women were mostly effective in using the postpartum methods. Doctors and nurses took place in the second plan. Important steps were taken to improve women's and children's health in our country. Consulting services which is a key element in family planning services is not at the desired level. Counseling skills of health professionals should be develop with an emphasis on antenatal care programs. Also these programs should take pregnant women together with their husbands. With this way desired goals can be reached in mother-child health in our country.

## Conflict of interest

We had no conflict of interest.
